# Joint-Sparing Resection of Juxta-Articular Primary Tumors of the Knee Using Titanium Alloy 3D-Printed Cutting Guides and Allograft Reconstruction

**DOI:** 10.3390/cancers16244185

**Published:** 2024-12-16

**Authors:** Maurizio Scorianz, Guido Scoccianti, Lorenzo Guariento, Monica Carfagni, Domenico Andrea Campanacci

**Affiliations:** 1 Orthopaedic Oncology and Reconstructive Surgery, Careggi University Hospital, 50134 Florence, Italy; guido.scoccianti@unifi.it (G.S.); lorenzo.guariento@unifi.it (L.G.); domenicoandrea.campanacci@unifi.it (D.A.C.); 2 Department of Industrial Engineering, University of Florence, 50139 Florence, Italy; monica.carfagni@unifi.it

**Keywords:** bone tumors, 3D printing, custom made, cutting guides, juxta-articular tumors

## Abstract

**Background:** Joint-sparing resection of juxta-articular knee tumors is challenging. With the development of 3D printing technology, custom-made cutting guides were introduced with the aim to improve the accuracy of tumor resection margins and to shape the allograft for adequate matching in biological reconstruction. **Materials and Methods:** Between December 2017 and July 2023, 13 patients underwent joint-sparing juxta-articular resection of the knee for a primary bone tumor at our institution. Resection was performed using a custom-made 3D-printed titanium alloy cutting guide and reconstruction was performed using a custom-shaped allograft. The mean follow up was 50 months (min 14–max 81). The study of cutting accuracy was performed by digital examination of CT images of the resection specimen in eight patients. **Results:** The average cutting error was 2.3 mm. Surgical margins were wide in all patients. Four patients developed postoperative complications that led to the removal of the allograft in one case. The average functional result according to the Musculoskeletal Tumor Society Score was 26 (min 14–max 30), and according to the Oxford Knee Score it was 44 (min 22–max 48). **Conclusions:** Custom-made titanium cutting guides resulted effective in improving the accuracy of joint-sparing juxta-articular resection of the knee and allograft matching.

## Simple Summary

The aim of our study was to assess the efficacy of 3D-printed titanium cutting guides to obtain precise tumor resection and allograft shaping in joint-sparing biological reconstructions around the knee. This paper presents the short- to medium-term results of patients treated with this technology, analyzing surgical, oncological and functional outcomes.

## 1. Introduction

Joint-sparing bone tumors resection of the knee represents a significant challenge in orthopedic oncology due to the complex anatomy of the region, which has a variable bone surface and due the presence of several insertions of ligaments and tendons. Therefore, to preserve the knee joint, juxta-articular or multiplanar osteotomies are often required.

In most cases, the tumor extension into the epiphysis does not allow for joint preservation and the most common treatment is to perform an intra-articular resection of the distal femur or proximal tibia and its replacement with a megaprosthesis. The sacrifice of the knee joint is counterbalanced by the relative simplicity and rapidity of the resection and reconstruction, early weight-bearing allowed after surgery and satisfactory functional outcomes. However, patients undergoing this kind of surgery are often young, and the prosthetic implant puts them at risk of subsequent revisions due to wear, loosening, mechanical failure or infection [1,2].

Recent advances in 3D printing technology have enabled new opportunities for more precise multiplanar resections by creating cutting guides customized for the specific patient’s anatomy. These cutting guides are commonly used to perform osteotomies in complex anatomical locations, such as the pelvis, or in osteotomies for the correction of limb deformities. With the same technology, it is possible to produce cutting guides specific for the allograft, used for biological reconstruction, making it possible to shape the allografts to adequately match the bone defect resulting from the tumor resection.

Titanium alloy, with its peculiar mechanical properties [3], was chosen by our team as an ideal material to produce these cutting guides. Its high modulus of elasticity allows for the creation of thinner guides, which are particularly useful in the complex anatomical region of the knee, where space is limited, and precision is paramount.

There are several articles in the literature regarding the use of cutting guides for the resection of bone tumors, but these are usually heterogeneous case series or single case reports.

The present study prospectively evaluated the outcome of 13 patients who underwent joint-sparing resection of a primary bone tumor of the knee and reconstruction with a custom-shaped massive allograft using a 3D-printed titanium alloy cutting guide.

We assessed the surgical margins, cutting accuracy, complications, oncological status and functional outcomes of patients.

## 2. Materials and Methods

Between December 2017 and July 2023, 13 patients underwent joint-sparing resection with allograft reconstruction for a primary bone tumor of the knee at our institution. The mean follow up was 50 months (min 14–max 81).

The tumor site was proximal tibia in 8 patients and distal femur in 5.

The histological diagnosis was adamantinoma in 3 patients, parosteal osteosarcoma in 4, peripheral chondrosarcoma in 3, low-grade central osteosarcoma in 1 and high-grade conventional osteosarcoma in 2. Ten patients were females and three were males. The mean age of patients at surgery was 34 years (min 14–max 68). All patients had localized disease at presentation.

The study was approved by the local ethics committee and written informed consent was obtained from participants.

Tumor extension was investigated by a preoperative contrast-enhanced MRI and CT scan of the bone segment. A CT-guided needle biopsy was performed in all patients to obtain histological diagnosis.

Patients affected by high-grade osteoblastic osteosarcoma received chemotherapy according to the ISG/OS-2 protocol [4] and underwent surgery 3 weeks after the final cycle. No patients underwent radiotherapy treatment.

Allografts were selected from the Tuscany Region (AOU Careggi) and the Emilia Romagna Region (IOR Bone Banks), matching their size with the patient’s anatomical segment using CT scan imaging. The average age of the donors at the time of death was 38 years (range 17–58).

**Figure 1:**
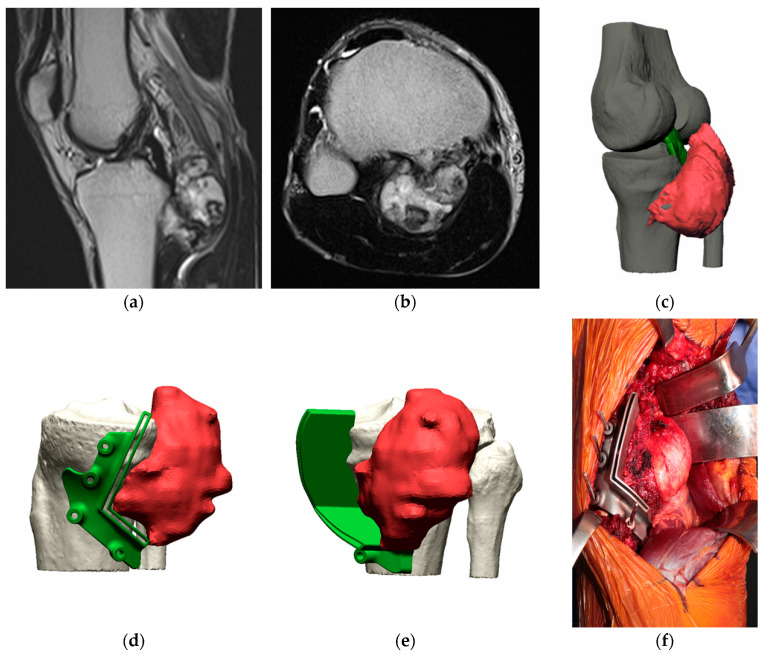

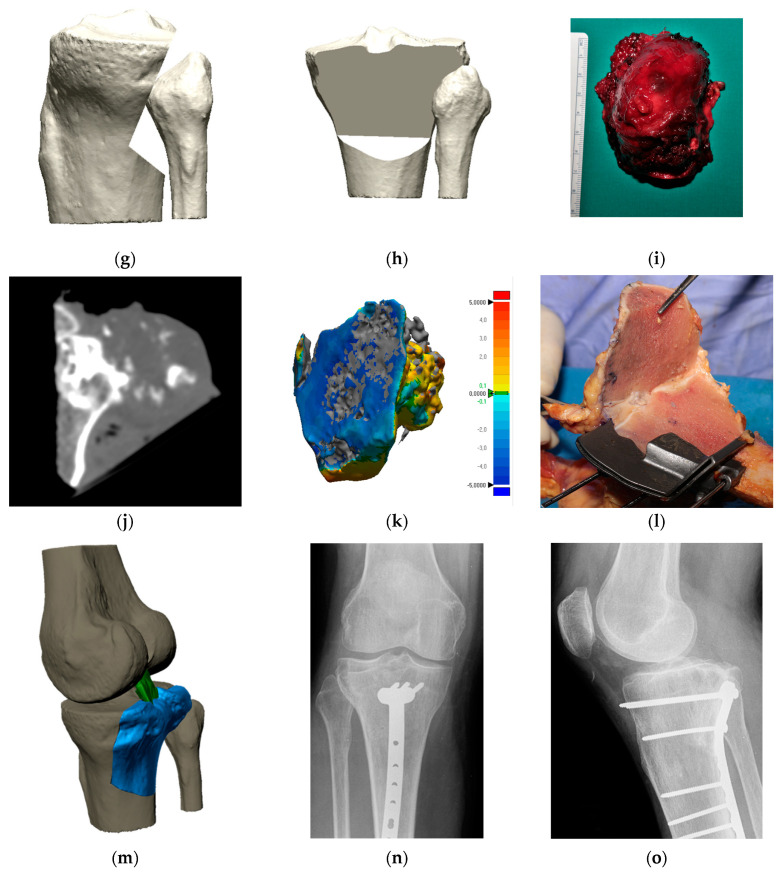
Treatment of a juxta-articular peripheral chondrosarcoma of the proximal tibia arising near the posterior cruciate ligament insertion (PCL). (**a**) Sagittal T1 weighted MRI showing the proximity of the tumor to the PCL insertion. (**b**) Axial T1 weighted MRI highlighting the origin of the tumor from the bony surface. (**c**) 3D model of the tumor (red) and of the PCL (green). (**d**,**e**) 3D model of the placement of the cutting guide around the tumor. (**f**) Application of the cutting guide to perform tumor resection, preserving the PCL insertion. (**g**,**h**) 3D model of the resection. (**i**,**j**) Specimen and CT scan of the specimen demonstrating wide margins. (**k**) Color map of the cutting errors of the specimen: the colors represent the difference between the preoperative surface of the digital planning and the surface of the resected bone. Green represents the ideal plane, while blue and red represent the osteotomy offset from the ideal plane. (**l**) Shaping of the allograft using an allograft-specific cutting guide to precisely fit the bone gap. (**m**) 3D model of the allograft reconstruction (blue). (**n**,**o**) Radiographs at one-year follow-up demonstrating integration of the allograft.

**Figure 2:**
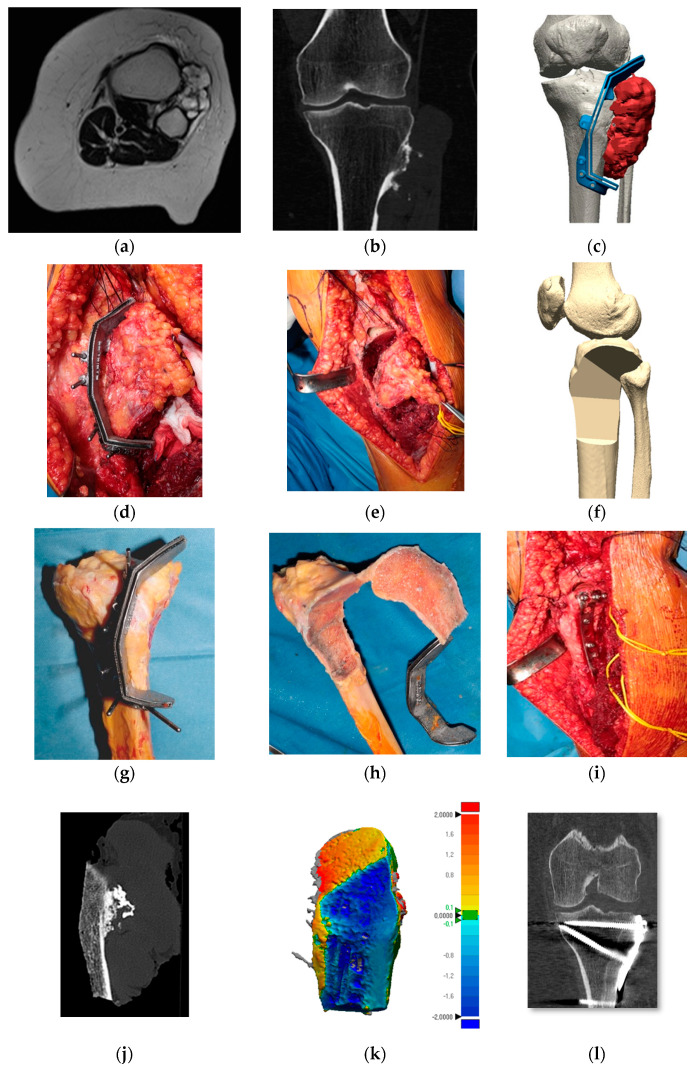
Treatment of a juxta-articular peripheral chondrosarcoma of the proximal tibia. (**a**) Axial T1 weighted MRI showing the tumor between the proximal tibia and fibula. (**b**) Tibial cortical erosion highlighted at the CT. (**c**) 3D model of the tumor (red) and of the cutting guide (blue). (**d**,**e**) Resection of the tumor. (**f**) 3D model of the resection. (**g**,**h**) Shaping of the allograft. (**i**) Allograft fixation. (**j**,**k**) CT scan of the specimen and color map of the cutting error. (**l**) CT scan showing allograft consolidation at 1-year follow-up.

After segmentation of the tumor (Mimics 24.0, Materialise NV Leuven, Belgium), a three-dimensional digital model of the anatomical region and tumor extension was created by fusion of CT and MRI images. The surgeon planned the osteotomy planes on the 3D model, taking care to maintain a wide margin but, at the same time, preserving as much as possible the healthy tissue and critical anatomical structures. Following these requirements, an engineer discussed with the surgeon the design of a patient-specific custom-made multiplanar cutting guide using computer-aided design (CAD) software (Geomagic Design X 2024.2, Oqton San Francisco, CA, USA), according to the extent of the tumor and the need to minimize soft tissue detachment and bone loss. According to the project, the cutting guide was printed and provided by Link Italia, Milan, Italy, MT Ortho Catania, Italy or Adler Ortho Milan, Italy. A different cutting guide was produced to shape the allograft to precisely match the bone defect resulting from the resection (Figure 1 and Figure 2). The material chosen to produce the cutting guides was titanium alloy (Ti 6Al 4V). Usually, the cutting guides employed in orthopedic surgery are made of polymer, because the cost of production and post-processing is lower than that of metal alloys [5]. However, the modulus of elasticity of titanium alloy is higher than that of polymer, allowing for the design of thinner cutting guides, reducing the need for soft tissue detachment [3]. This is crucial in the knee area where multiplanar joint-sparing resections can be performed preserving tendinous insertions.

The mean production time for the creation of the cutting guides was 12 days (min 7–max 15). This time included allograft research, image segmentation, cutting guide design, production and shipping.

The mean number of cutting planes featured on the cutting guides was 4 (min 2–max 8). The number of cutting slots, i.e., multiplanar cuts, was 6 in three patients, 5 in one, 4 in four, 3 in one and 2 in four patients.

Prior to surgery, the cutting guides were subjected to steam sterilization in an autoclave at 134 °C for 5 min at a pressure of 3 bar.

To avoid metal debris due to the saw oscillation, a special saw was used whose oscillating part is located exclusively at the tip (Falcon Precision, Stryker Corporation, Kalamazoo, MI, USA).

## 3. Results

Joint-sparing resections were performed with multiplanar osteotomy of the distal femur in five cases and proximal tibia in eight cases. Juxta-articular resections at less than 2.5 cm from the joint line were reconstructed with a hemicortical allograft in four cases (3 tibia, one femur) and a hemiarticular allograft in two cases (tibia). Resections between 2.5 cm and 5.5 cm from the joint line (mean 3.7 cm, median 3 cm) were reconstructed with an intercalary allograft alone in four cases (three femurs, one tibia) and with an allograft associated to a vascularized fibula in three cases (two tibia, one femur) [6].

### 3.1. Surgical Margins and Accuracy

Wide (R0) margins were obtained in all patients.

The study of cutting accuracy was performed on eight patients by digital examination of CT images of the resected specimen (Figure 1k and Figure 2k). This method provides an accurate measurement and does not expose the patient to ionizing radiation. The osteotomy planes designed with the preoperative 3D planning were compared with the final cuts of the resected surgical specimen.

A total of 27 cutting planes were evaluated for accuracy in eight surgical specimens.

The median cutting error was found to be 2.3 mm and mean cutting was 1.5 mm, with a minimum error of 0 mm and a maximum error of 7 mm.

Out of a total of 27 cutting planes analyzed by CT, only one cut presented an error of 7 mm, due to the particular complexity of the positioning of the cutting guide. Nine resections (33%) presented an error between 5 and 2 mm. The remaining 17 cutting planes (63%) presented an error of less than 2 mm.

### 3.2. Oncological Status at Latest Follow Up

No local recurrence or distant metastases were observed; all patients were disease-free at latest follow-up.

Histologic examination of the two high-grade osteoblastic osteosarcomas showed 95% and 75% necrosis, corresponding to grade 3 and 2 of the Tumor Necrosis Grading System [7], and both patients received adjuvant chemotherapy.

### 3.3. Complications

Four patients (30%) developed postoperative complications that resulted in allograft removal in one case (7.7%).

One patient developed nonunion at the proximal femoral osteotomy which was successfully treated with autologous bone graft harvested from the iliac crest 10 months after the initial surgery.

Another patient developed nonunion at the distal tibial osteotomy with hardware failure 12 months after intercalary resection and reconstruction with an intercalary allograft. The patient was treated with a new plate fixation and autologous bone graft harvested from the iliac crest, achieving union after 3 months.

One patient suffered a displaced fracture of the femur at distal osteotomy 4 months after multiplanar resection and hemicortical allograft reconstruction. After new osteosynthesis and autologous bone grafting, a new fracture was observed and the patient eventually underwent distal femur resection and reconstruction with megaprosthesis at 10 months from primary surgery.

One patient had a vein thrombosis of the vascularized fibula graft, detected by congestion of the pedicled skin island, that required a new microsurgical anastomosis the day after surgery.

### 3.4. Functional Results

Functional score was assessed at the last follow-up using the revised score of the Musculoskeletal Tumor Society (MSTS) [8]. The maximum score is 30 points and for the lower extremities it is evaluated according to the following categories: pain, function, emotional acceptance, use of external support, functional independence and gait. One patient who had a megaprosthesis implanted due to complications was excluded from the functional evaluation.

The Mean Musculoskeletal Tumor Society Score (MSTS) was 27/30 (maximum 30, minimum 14, median 29); the mean Oxford Knee Score (OKS) was 44/48 (maximum 48, minimum 22, median 47); the lowest scores were recorded in a patient who had signs and mild symptoms of knee osteoarthritis before surgery, which increased at follow up.

## 4. Discussion

The knee is the most common site of malignant bone tumors [9]; the usual surgical treatment consists in the resection of the tumor with sacrifice of the knee joint and its replacement with a megaprosthesis [10]. This relatively quick procedure allows for rapid functional recovery of the patient. However, in the long term, prosthetic reconstructions are burdened by the risk for new surgeries due to infection, loosening and mechanical complications [2]. On the other hand, biological reconstructions are more technically demanding but they have shown to maintain a good functional outcome in the long term once early complications are overcome [11,12,13,14].

The aim of our study was to assess the efficacy of 3D-printed titanium cutting guides to obtain precise tumor resection and allograft shaping in joint-sparing biological reconstructions around the knee and to evaluate the functional results and complications in the short term.

Cutting guides have been used in a number orthopedic indications, such as deformity correction and arthroplasty [15,16]. Their accuracy has been evaluated for bone tumor resections in both simulated and real settings.

Vamiq et al. [17] simulated the resection of a distal femur tumor with cutting guides using sawbones. The resection was performed by 10 experimenters with no experience in using cutting guides. The mean deviation error reported ranged between 2.86 mm and 6.54 mm.

Khan et al. [18] found a maximum deviation of 9 mm in manual resection and 2 mm in resection with cutting guides on six matched pairs of cadaveric femurs. The average deviation was 3.1 mm in the manual resections and 0.8 mm in the cutting guide resections.

In another cadaveric study of eight tibias and eight femurs, Bosma et al. [19] highlighted the greater accuracy of computer-assisted resections or cutting guides compared to freehand cuts. Both assisted techniques allowed for a location accuracy of less than 2 mm.

Dong et al. [20] described the use of cutting guides for resection in several skeletal segments, seven of which were the femur and tibia in patients with primary bone tumors. In these localizations, the average difference between planned and resection length was 8.35 mm.

Schweizer et al. [21] described a technique consisting of patient-specific instruments with drill sleeves obtaining osteotomy planes by perforating the bone with consecutive drills instead of using a saw blade. The cohort of this study [22] included two resections of distal femur and one of proximal tibia, using this technique. The histological diagnosis of the three patients was osteochondroma, chondrosarcoma, and low-grade osteosarcoma. Cutting accuracy was measured directly on the specimen with pathologic analysis in two cases or by postoperative CT scan of the specimen in one case. The cutting error was of 1.33 and 0.74 mm in the two patients who underwent a resection through a simple plane and of 2.4 mm in the patient who had a complex multiplanar osteotomy.

In a series of resections performed with cutting guides of tumors from different bone segments, Woong reported the resection of a periosteal osteosarcoma of the distal femur, with a cutting accuracy of 1 mm measured on the specimen pathologic analysis [23].

Bellanova et al. [24] presented a series of four patients who underwent resection with a polymer cutting guides for tibia tumors (three osteosarcoma, one Ewing sarcoma) and allograft reconstruction. Three were intercalary resections, one osteoarticular. An evaluation of the cutting error was not reported in the study; however, the minimum planned margin was 3.5 mm, and all patients presented free margins at histopathologic follow-up.

The mean cutting error in our series was 2.3 mm and all resections had wide margins.

The error measurement in our study was performed on the 3D model of the resected bone, which we considered to be the most accurate and reliable method for accurately studying resection geometry. It is worth noting that a direct comparison of the resection error between different studies is difficult because studies often do not report the measurement method used, while others perform the measurement physically directly on the specimen [22,23,24].

The lower accuracy compared with cadaveric studies could be the result of the higher number of multiplanar cuts present in the cutting guides used in our study and by the greater difficulty of positioning the cutting guides in intraoperative setting where, differently from cadavers, it is necessary to preserve the soft tissues insertions as much as possible, eventually hampering a perfect cutting guide positioning on the bone surface.

This highlights the importance of a strict collaboration in planning between the surgeon and the bioengineer, because the location and shape of the cutting guides must consider not only the oncological margin but also the surgical approach and the realistic ability to achieve an adequate intraoperative contact between the guide and bone.

Nonetheless, the entity of cutting errors in our series never led to an inadequate surgical margin, with all procedures presenting wide histological margins. Considering the range of cutting errors, a surgical margin of at least 10 to 15 mm must be considered when planning the procedure, the shape and position of cutting guides.

In many studies, the method of error study includes examination of the anatomical piece, or in some studies the method used is not reported, thus making it impossible to compare the errors.

In the literature, nonunion, fracture and infection are the most common complications after an allograft reconstruction [25,26].

Two patients in our series developed a complication. One suffered a nonunion, successfully treated by augmentation with autologous bone grafts from the iliac crest, while the second patient developed a host bone fracture that required distal femur resection and reconstruction with megaprosthesis after a failed biologic salvage attempt with new synthesis and autologous bone grafts.

In their study of 118 host–donor junctions, in relation with the type of anatomical region and type of internal fixation, Muscolo et al. [26] performed a comparison of allograft reconstructions with plate and screws fixation and found 8% of nonunion in the diaphyseal region (3 out of 39 patients) and no cases in the metaphyseal region (22 patients). Accordingly, in our series, the only nonunion occurred in the diaphyseal region.

In a review of osteotomies with the employment of cutting guides, Aiba et al. [27] compared the different materials that can be used to produce them. The use of metal represents a greater complexity and cost of printing, requiring the more complex powder bed fusion printers [5]. On the other hand, this study concludes that metallic cutting guides present greater hardness and their sterilization can be more simple and less expensive since they can be subjected to autoclave temperatures without losing their properties.

In our study, the use of titanium allowed for the creation of thin cutting guides that required minimal soft tissues detachment. We do not have a control group and therefore we lack data of comparison between different techniques, but we think that it can be legitimate to suggest the use of these devices in juxtarticular locations, where a guide made of nonmetallic material can be too bulky.

Our study has several limitations. First, the relatively small number of patients and short-term follow-up are notable constraints. Primary bone tumors are rare, and only a minority present with characteristics which make it possible to perform a joint-sparing juxta-articular resection. Additionally, the widespread use of 3D printing technology in orthopedic oncology has only emerged in the past decade, limiting the available patient pool. The small sample size of only 13 patients means that the results, while promising, are not robust enough to be generalized without further validation in larger cohorts.

The mean follow-up duration of 50 months is relatively short to assess reconstruction outcome in the long term. Longer follow-up data will be essential to prove the long-standing validity of this technique. Another limitation is the absence of a control group of patients treated with megaprostheses, which would provide a valuable comparison.

## 5. Conclusions

This study demonstrates that joint-sparing resection of juxta-articular primary tumors of the knee using 3D-printed titanium alloy cutting guides, followed by reconstruction with custom-shaped allografts, is a viable surgical approach that combines oncological efficacy with functional preservation. Although in our series the short-term complications rate was relevant, the overall outcome in terms of oncological control and survival of the biological reconstruction was successful.

## Data Availability

The raw data supporting the conclusions of this article will be made available by the authors on request.
